# Metabolic PCTA-Based Shift Reagents for the Detection of Extracellular Lactate Using CEST MRI

**DOI:** 10.1021/jacsau.4c01020

**Published:** 2025-02-10

**Authors:** Remy Chiaffarelli, Pedro F. Cruz, Jonathan Cotton, Tjark Kelm, Slade Lee, Mohammad Ghaderian, Max Zimmermann, Carlos F. G. C. Geraldes, Paul Jurek, André F. Martins

**Affiliations:** † Werner Siemens Imaging Center, Department of Preclinical Imaging and Radiopharmacy, University Hospital Tübingen, Tübingen 72076, Germany; ‡ Cluster of Excellence iFIT (EXC 2180) “Image-Guided and Functionally Instructed Tumor Therapies”, University of Tübingen, Tübingen 72076, Germany; § German Cancer Consortium (DKTK), Partner Site Tübingen, German Cancer Research Center (DKFZ), Im Neuenheimer Feld 280, Heidelberg 69120, Germany; ∥ Coimbra Chemistry Center-Institute of Molecular Sciences (CQC-IMS), Faculty of Science and Technology, University of Coimbra, Coimbra 3004-535, Portugal; ⊥ Macrocyclics, Inc., Plano, Texas 75074, United States; # CIBIT—Coimbra Institute for Biomedical Imaging and Translational Research, University of Coimbra, Coimbra 3000-548, Portugal; ¶ Department of Life Sciences, University of Coimbra, Coimbra 3000-456, Portugal

**Keywords:** lactate, PARACEST, ytterbium, europium, CEST MRI

## Abstract

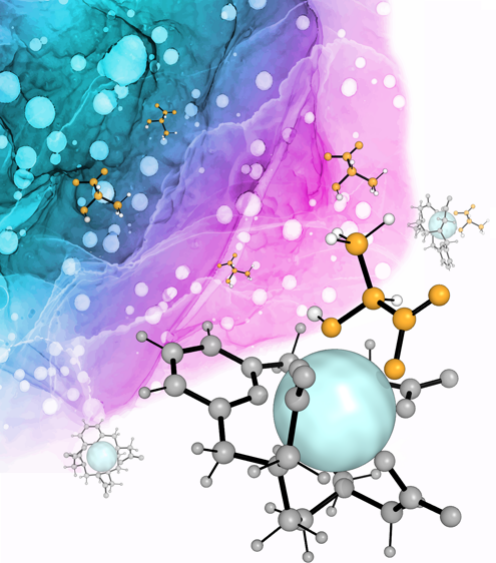

Lactate is a key metabolic driver in oncology and immunology. Even in the presence of physiological oxygen levels, most cancer cells upregulate anaerobic glycolysis, resulting in abnormal lactate production and accumulation in the tumor microenvironment. The development of more effective, sensitive, and safe probes for detecting extracellular lactate holds the potential to significantly impact cancer metabolic profiling and staging significantly. Macrocyclic-based PARACEST agents have been reported to act as shift reagents (SRs) and detect extracellular lactate via chemical exchange saturation transfer (CEST) MRI. Here, we introduce a new family of SRs based on the PCTA ligand, an inherently stable and kinetically inert group of molecules with the potential for (pre)clinical translation. We observed that Yb-PCTA and Eu-PCTA can significantly shift lactate –OH signals in the CEST spectra. *In vitro*, CEST MRI experiments proved that imaging extracellular lactate specifically with these complexes is feasible even in the presence of competing small metabolites in blood and in the tumor microenvironment. *In vivo* preclinical imaging showed that Yb-PCTA can be safely administered intravenously in mice to detect extracellular lactate noninvasively. This work contributes to the field of precision imaging in medicine and provides evidence that the PCTA-ligand is a valuable scaffold for developing molecular and metabolic imaging sensors.

## Introduction

Metabolic imaging, the noninvasive visualization of metabolite distribution within living organisms, is essential for understanding disease processes and assessing treatment responses. It enables the early detection, characterization, and monitoring of relevant diseases, e.g., in oncology and neurology, and therefore holds paramount significance in medicine and biomedical research. A key aspect of metabolic imaging is that it allows monitoring of the dynamic behavior of cells and tissues, offering the potential to identify abnormal metabolic patterns associated with disease states. For example, cancer cells overproduce lactate, even in the presence of high oxygen and glucose levels, a phenomenon called the Warburg effect. Most of the lactate produced by cancer cells builds up in the extracellular environment, affecting the tumor and the homeostasis of the surrounding tissues.^1^ Detecting the lactate buildup in tissues is thus critical as it translates to a precise measure of cancer metabolic activity and may provide a direct readout of cancer staging.

Modern imaging technologies offer substantial promise for assessing cancer metabolic activity and tumor staging. Magnetic resonance spectroscopy, aided by hyperpolarized ^13^C-labeled substrates like pyruvate^2^ and fumarate^3^ or deuterated metabolites like glucose and acetate,^4^ provides invaluable information regarding enzymatic activity in tumors related to staging and therapy response. These methods, however, lack spatial resolution and cannot distinguish between intra- and extracellular metabolic processes, preventing the accurate quantification of extracellular lactate produced by the tissues. The chemical shift between lactate hydroxyl and water protons allows the detection of lactate with chemical exchange saturation transfer (CEST) techniques;^5,6^ however, it does not aid in the discrimination between extra- and intracellular lactate. The water signal obscures the slight chemical shift of free lactate (<1 ppm), hampering the quantification of small lactate concentrations. Hence, a method for noninvasively imaging extracellular lactate produced by the cancer cells is of utmost importance to better understand metabolic dynamics and compartmentalization.

Recently, a family of shift reagents (SRs) has demonstrated remarkable capabilities in selectively detecting extracellular lactate using CEST. These CEST SRs consist of stable inorganic complexes formed by the complexation of lanthanide ions with DOTA-type macrocycles. A Yb^3+^ complex based on a derivative of DO3A, Yb-MBDO3AM, was reported to bind to lactate at different concentrations.^7^ A simplified version of these SRs utilized the Eu-DO3A complex, which coordinates with lactate by displacing the two water molecules directly bound to the inner sphere of the lanthanide.^8^ This specific interaction creates a ternary complex with lactate, allowing for the precise detection of the lactate –OH group, effectively shifting it away from the bulk water signal. When coadministered in the ternary complex with lactate, Eu-DO3A could readily detect extracellular lactate excreted in the bladder of a healthy mouse. Despite detecting the intact Eu-DO3A form in the urine, lanthanide complexes formed with DO3A show a lower kinetic inertness than most octacoordinated Ln-DOTA complexes. Kinetic inertness is a crucial parameter when designing medical imaging inorganic contrast agents (CAs) directed to clinical translational applications as the release of lanthanide ions (e.g., Gd^3+^) may lead to severe physiological complications associated with nephrogenic systemic fibrosis (NSF) in patients with renal impairment.^9^ In this pursuit, the pyclen-based PCTA ligand proved to be an important alternative to cyclen-based DOTA-type macrocycles due to its high thermodynamic stability (log *K*_ML_ = 18.15–20.63 for Ce–Yb) and kinetic inertness (*t*_1/2_ in the range of hours) similar to those of Ln-DOTA complexes.^10^ Like Ln-DO3A complexes, Ln-PCTA complexes generally show higher hydration (*q* = 2) and faster water exchange rates (*k*_ex_) than Ln-DOTA complexes due to their increased rigidity akin to Ln-DO3A.^11−15^ The heptadentate PCTA ligand forms Ln^3+^ complexes that are highly suitable for safe translational applications, despite the increased hydration of Ln-PCTA complexes, which allows other biogenic molecules to replace the water molecules in the coordination sphere. In 2022, the FDA approved the Gd-PCTA-based complex (gadopiclenol) as a new MRI CA. This CA shows improved *T*_1_ MR contrast properties compared to traditional Gd-based MRI CAs with octadentate ligands.^16^

Here, we describe the development of a new generation of SRs based on Ln-PCTA for the selective detection of extracellular lactate by CEST MRI. For this purpose, we selected Ln^3+^ ions that have a minor impact on the *T*_1_ relaxation of water protons in the concentration ranges relevant for CEST MRI: Eu^3+^, Yb^3+^, and Pr^3+^.^17^ The interaction between Ln^3+^-based PCTA complexes (Yb-PCTA, Eu-PCTA, and Pr-PCTA) and lactate was characterized by using high-resolution nuclear magnetic resonance (NMR), revealing the formation of lactate-Ln-PCTA ternary complexes. The CEST effect of these complexes was quantified *in vitro* at 7T, demonstrating a specific signal for lactate. Additionally, binding affinity values indicated a weak interaction, suggesting that lactate was not significantly depleted from the bloodstream when these SRs were used for *in vivo* imaging. *In vitro* cytotoxicity assessment validated the safety and feasibility of SRs for *in vivo* imaging. A preclinical animal study using Yb-PCTA showed the safe detection of the lactate-Yb-PCTA ternary complex in the bladder, emphasizing the translational potential of this approach. Moreover, the shiftCEST approach was validated in a tumor-bearing animal model, further showcasing its ability to monitor lactate metabolism and tumor microenvironment (TME) characteristics. This work highlights the capability of lanthanide-based PCTA complexes and CEST MRI for metabolic imaging applications, offering a new tool for profiling various cancer cell lines and the associated TME.

## Results and Discussion

**Figure 1:**
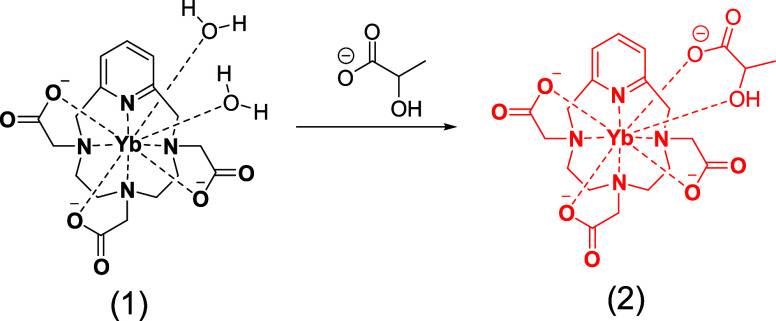
Illustrative schematic of the formation of a ternary complex lactate-Yb-PCTA.

In this study, we investigated whether a new generation of SRs based on Ln-PCTA could detect extracellular lactate and provide selective detection of extracellular lactate by CEST MRI *in vitro* and preclinically. We prepared several solutions containing Ln-complexes (Ln: Eu^3+^, Yb^3+^, and Pr^3+^) to explore the potential formation of ternary complexes between Ln-PCTA and lactate. Phantom imaging experiments were performed in serum to mimic physiological conditions and at pH 6 and 7 to more closely resemble acidic conditions often found in the TME, where lactate accumulates.^18^ Eu^3+^, Yb^3+^, and Pr^3+^ were selected based on the expected induced chemical shifts resulting from their large magnetic moments^19^ and their limited impact on the longitudinal and transversal relaxation of water protons (*r*_1_, *r*_2_) compared to other Ln^3+^ such as Gd^3+^. The PCTA complexes employed in the study were synthesized as described in the Supporting Information. As depicted in Figure 1, a ternary complex can be formed between the Ln-PCTA complexes and lactate by replacing at least one of its two inner-sphere water molecules, as observed before between Ln-DO3A-type complexes with lactate and other α-hydroxy-carboxylates.^8,20^ To determine if the Ln-PCTA complexes are selective for the extracellular fraction of lactate, we tested whether Ln-PCTA complexes could cross cellular membranes using a permeability artificial membrane penetration assay (PAMPA).^21^ The results showed only trace levels of Ln-PCTA complexes in the acceptor compartment after 21 h (9.5 ± 0.9% of the theoretical equilibrium concentration) (Figure S1A). Additionally, in a cell experiment, the growing medium of cells incubated with Yb-PCTA or a known extracellular CA (Gd-DOTA, Gadovist) was analyzed with bulk magnetic susceptibility (BMS) NMR measurments to determine the concentration of the Ln^3+^ complexes after 2 h of incubation and quantify the uptaken fraction. No significant difference was found in the concentration of wells containing cells and control wells, indicating that these complexes primarily remain extracellular and are imported in the intracellular compartment only to a minor extent (Figure S1B). This makes them ideal for detecting extracellular lactate, with negligible intracellular interference in the timespan that could be useful for *in vivo* imaging (15–30 min).

**Figure 2:**
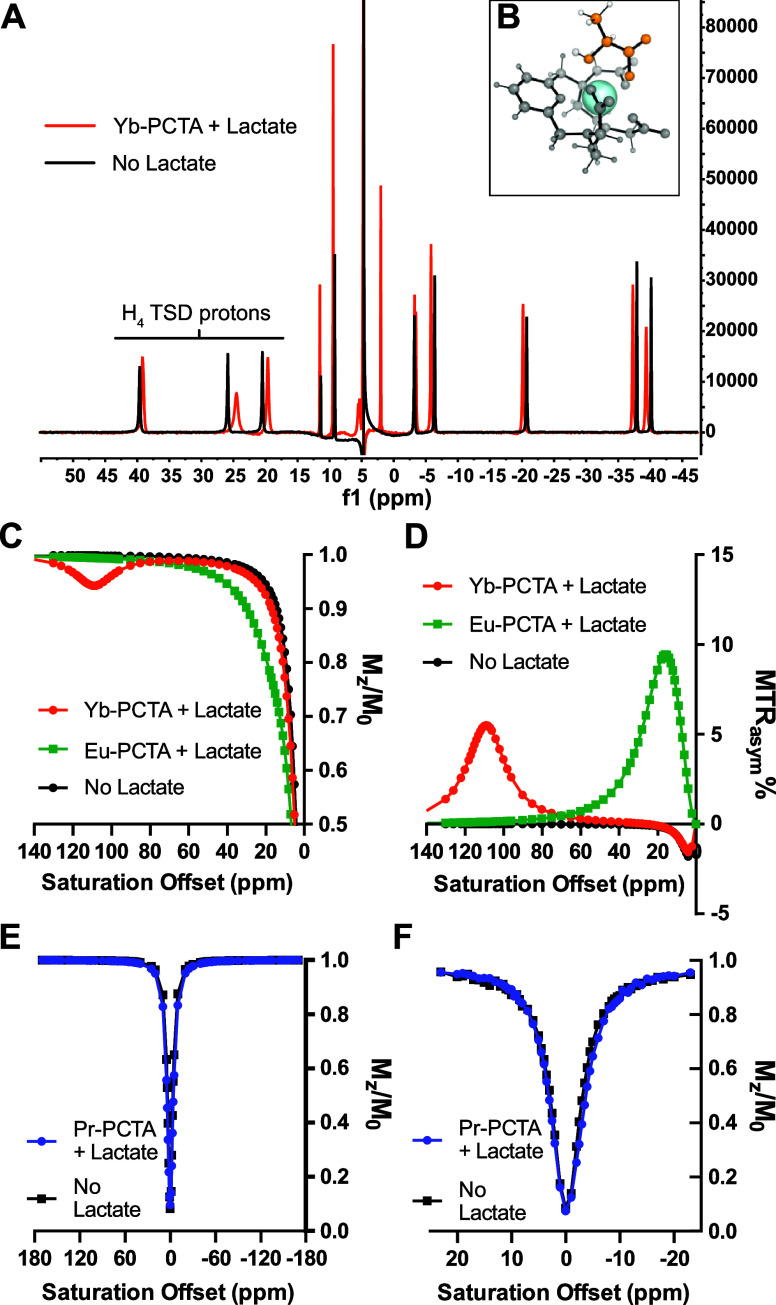
(A) ^1^H NMR spectra of a solution containing 3 mM Yb-PCTA (black line) and 2:1 lactate-Yb-PCTA mixture (orange line), acquired at 400 MHz, 363 K, pH 7.1. (B) 3D schematic of the interaction lactate-Ln-PCTA. (C,D) z-spectra (C) and MTRasym % (D) plot of 50 mM Yb- and Eu-PCTA in HEPES buffer with 50 mM lactate, acquired at 7T MR after presaturation pulses of 16 μT and 5 s, at 298 K, pH 6.7. “No lactate″: spectrum of Eu-PCTA without lactate. CEST spectra were fitted to Lorentzian-line shapes using a two-pool model (lactate-Ln-PCTA and water). (E) z-spectra of 1:1 20 mM lactate-Pr-PCTA or 20 mM lactate, acquired with 5 s 16 μT saturation pulses, pH 6. (F) z-spectra of 1:1 40 mM lactate-Pr-PCTA or 40 mM lactate, acquired in a narrower spectral bandwidth with 5 s 20 μT saturation pulses, pH 6.

In an aqueous solution, DOTA-type complexes show the presence of twisted square antiprismatic (TSAP) and square antiprismatic (SAP) diastereoisomeric pairs of enantiomers, which result from the combination of the two square [3,3,3,3] conformations of the cyclen macrocyclic ring, in which the four ethylenediamine N–C–C–N bridges adopt the identical gauche conformations of opposite helicities, (δδδδ) or (λλλλ), with the two possible orientations of the acetate arms attached to the macrocyclic nitrogen atoms, Λ (clockwise) and Δ (counterclockwise).^22^ Eu- and Yb-DO3A were reported to show a preferred SAP structure, with the metal-coordinated lactate having the OH group in an apical position and showing highly shifted CEST peaks of approximately 45 and 150 ppm, respectively.^8,20^ A less favored TSAP structure produced a smaller shift (16 ppm) for the CEST peak of Eu-DO3A-lactate with the OH group in a more equatorial position.^8^ However, in the case of Ln-PCTA, the rigidity of the pyridine group in the pyclen ring makes it impossible to adopt the [3,3,3,3] conformation. Instead, it adopts a [4,2,4,2] conformation bisected by a mirror plane with the nitrogen atoms located in the center of each side of the ring and the three ethylenediamine N–C–C–N bridges having (δλδλ) conformations. The Ln-PCTA complexes feature three acetate arms and can adopt twisted snub disphenoid (TSD) coordination geometries. A 3D structure in Figure 2B shows the proposed interaction between Ln-PCTAs and lactate. These geometries exhibit both (δλδλ)Λ and (δλδλ)Δ*o*rientations, and due to the rigidity of the pyridine unit, the four nitrogen atoms are not coplanar. Consequently, the TSAP and SAP conformations typical of DOTA-like complexes are unattainable.^23,24^ Taking this information into account, we determined the diastereoisomers of Ln-PCTA-lactate in solution using high-resolution ^1^H NMR spectra of Yb-, Eu-, and Pr-PCTA in the absence/presence of 2 equiv of lactate at various temperatures and pH values. The Ln-PCTA complexes are highly fluxional at high temperatures, with a fast exchange in the NMR timescale at 90 °C, between the dominant TSD (δλδλ)Λ isomer (10 resonances, as expected from the structure) with the very low populated (<10%) TSD (δλδλ)Δ isomer, leading to extensive broadening of most resonances at 25 °C.^10^ In the presence of lactate, the ^1^H NMR spectra at 90 °C showed two extra signals (2.01 and 5.42 ppm for Yb-PCTA-lactate) corresponding to the CH_3_ and CH groups of coordinated lactate in the Ln-PCTA-lactate ternary complexes in solution, which displayed only the TSD (δλδλ)Λ isomer. Again, this isomer is in fast exchange with the very minor (δλδλ)Δ isomer, as reflected in an extensive broadening of most resonances at lower temperatures (Figures 2A, S2–S4) and the presence of exchange cross-peaks in the EXSY spectrum of Eu-PCTA different from those of the COSY spectrum (Figure S5).^20^ The resonances of bound lactate exhibit some broadening at 90 °C compared to the free lactate resonances. However, as temperatures decrease, they gradually broaden until disappearing entirely at ∼0 °C, suggesting a fast-to-intermediate exchange regime in their binding to Ln-PCTA (Figures S4 and S5). This suggests a relatively weak interaction between lactate and the Ln-PCTA complexes, a finding supported by CEST titrations (Figure S6 and Table 1). This weak interaction may be attributed to the TSD conformation of the complex, where the four nitrogen atoms of the pyclen ring do not align in the same plane—two are positioned above and two below the median plane. Consequently, the three Ln-bound carboxylate oxygens are located slightly off the single plane. This distortion of the two available lactate-binding positions within Ln-PCTA could lead to steric hindrance in lactate binding. Additionally, a recent report shows how a 100-fold excess of lactate induces a 25% decrease of the *r*_1_ of Gd-PCTA, confirming the meager binding.^25^ While the axial CH_2_ protons from the macrocyclic ring in PCTA complexes showed less shifted resonances compared to the corresponding DO3A complexes, typical of TSAP structures, we observed shifts for all the proton resonances and broadening of some of them when lactate was bound. Hence, we anticipated a reasonable –OH CEST shift in the CEST spectra for the lactate –OH group as it exchanges with the surrounding bulk water. However, for efficient CEST imaging, the *T*_1_ relaxation times of the exchanging system must be sufficiently long to ensure that the CEST signal does not decrease rapidly during the acquisition.^26^ We have determined *T*_1_ values and the corresponding *r*_1_ relaxivity, a key governing parameter for CEST detection, in blood serum at 37 °C. We measured *r*_1_ values of 0.009 mM^–1^·s^–1^ for Yb-PCTA, 0.003 mM^–1^·s^–1^ for Eu-PCTA, and 0.007 for mM^–1^·s^–1^ Pr-PCTA (Figure S7). Therefore, the impact of longitudinal relaxation on CEST detection can be considered negligible.^27,28^

**Table 1:** Binding and Exchange Rates of Lactate·SRs^a^

	pH	*K*_A_ (M^–1^)	*k*_ex_ (s^–1^)
Yb-PCTA	6	20 ± 2	1270 ± 35
	7	11 ± 1	1770 ± 40
Eu-PCTA	6	100 ± 10	1810 ± 45
	7	25 ± 3	2820 ± 90
Yb-DO3A	6.5	N/A	1900^b^
Eu-DO3A	6	45^d^	2470^c^
	7	42^d^	2180^c^

a Binding affinities (*K*_A_) and exchange rates (*k*_ex_) for Yb- and Eu-PCTA were determined as described in Supporting Information.

b Determined at 298 K at 9.4 T by titrating B_1_ from 2.34 to 23.4 μT by Zhang et al., 2017.^100^

c Determined at 298 K at 9.4 T by titrating B_1_ from 2.35 to 23.5 μT by Zhang et al., 2017.^8^

d Determined by fitting CEST titrations collected using a B_1_ of 23.5 μT by Zhang et al., 2017.^8^

We performed CEST MRI experiments in phantoms containing one of the three Ln-PCTA complexes in the absence and presence of lactate at different concentrations. CEST signals originating from lactate were readily detected by using a 7T MR scanner for Yb and Eu complexes (Figure 2C,D). However, Pr-PCTA (20 and 40 mM) did not show a significant CEST effect in the presence of lactate (Figure 2E,F). In the presence of one lactate equivalent, Yb-PCTA produces a prominent and distinct CEST peak at around 109 ppm, compared to the typical 0.6–0.8 ppm for noncoordinated lactate. Meanwhile, the shift in the CEST signal induced by Eu-PCTA was slightly smaller than that previously reported with Eu-DO3A (approximately 14 ppm). The effect is even more evident when a lower saturation power (8 μT) is used thanks to a sharper definition of the water peak and reduced magnetization transfer (MT) effects (Figure S8). The quantification of the CEST effect, represented by the MT ratio asymmetry percentage (MTR_asym_ %), illustrates a significant and substantial CEST signal arising from the lactate·Eu-PCTA and lactate·Yb-PCTA ternary complexes, as illustrated in Figure 2D. This robust CEST effect observed with the ternary complexes sharply contrasts with the negligible CEST response observed with Eu–Yb–PCTA alone. We hypothesize that the lack of a detectable CEST effect with Pr-PCTA is likely due to the rapid proton exchange rate (*k*_ex_) between the –OH group of lactate and bulk water. For optimal CEST detection, the proton exchange rate should be within the slow exchange regime, where the frequency difference (Δδ) between the exchanging sites is greater than the exchange rate (Δδ > *k*_ex_).^29^ In this case, the faster exchange kinetics may shift the system out of this slow exchange regime, making it difficult to observe a clear CEST signal. In a previous report, the –OH protons of a Pr^3+^ complex produced a CEST effect at −25.2 ppm with a *k*_ex_ of 3.5 × 10^3^ s^–1^.^30^ Assuming a similar *k*_ex_ for lactate-Pr-PCTA, the absence of a clear CEST peak could be attributed to a chemical shift smaller than the *k*_ex_. A small chemical shift is likely due to a weaker pseudo-contact shift contribution associated with the ligand field stabilization of this complex. This diminished chemical shift reduces the separation between the labile proton signal and the bulk water, further hindering the detection. The *k*_ex_ for Yb- and Eu-PCTA, calculated using the Omega plot method at pH 6 and 7,^31^ were generally slower when compared to the values previously reported for DO3A-based SRs. Interestingly, both SRs exhibited a noteworthy minimum *k*_ex_ at pH 6, suggesting a similar acid-based proton catalytic effect (Table 1). The lower *k*_ex_ allows the application of relatively modest saturation powers (B_1_ < 12 μT) to detect the CEST effect. This represents a clear advantage for *in vivo* translational studies, where lower saturation powers are generally preferred.

**Figure 3:**
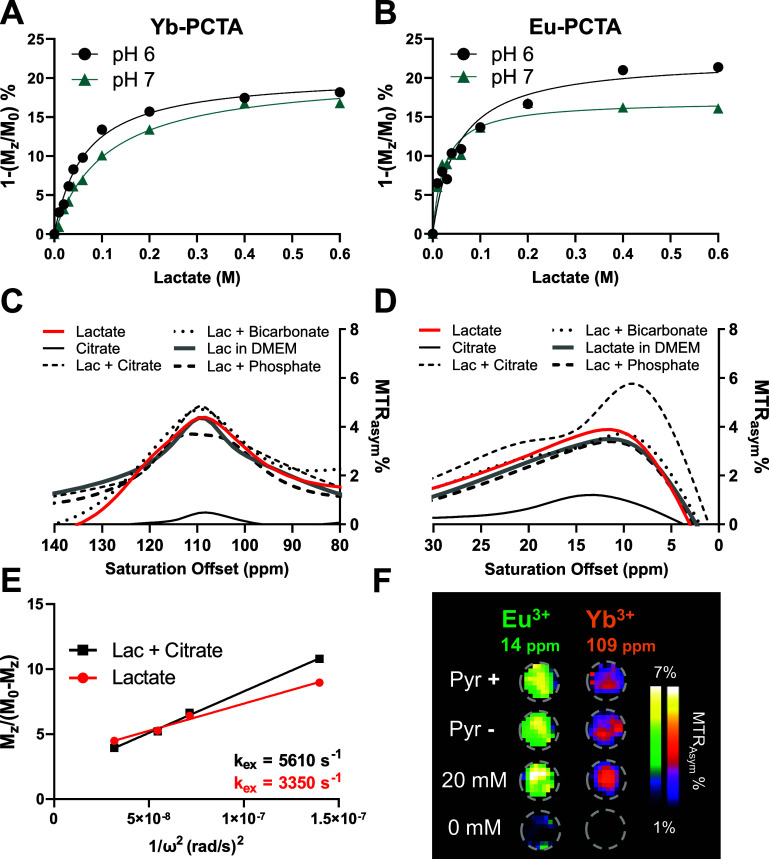
(A,B) Effect of lactate titration (0–0.6 M) on the CEST effect produced by 20 mM Yb-PCTA (A) or Eu-PCTA (B) at pH 6 and 7. Lines = fitting line. CEST effect at 109 ppm (Yb-PCTA) and 14 ppm (Eu-PCTA) were obtained at 298 K after a presaturation of 21 μT, 5 s. (C,D) MTR_asym_ % plot of solutions containing 20 mM Yb-PCTA (C) or Eu-PCTA (D) and 40 mM lactate, citrate or different combinations of lactate and other metabolites and DMEM, pH 6, 298 K. (E) Omega plot of Eu-PCTA with lactate or lactate and citrate (Lac + citrate). (F) CEST images of MC-38 colon adenocarcinoma cell growth media collected after 48 h of culture with or without 1 mM pyruvate, mixed with 20 mM Yb-PCTA or Eu-PCTA.

To better understand the strength of the interaction, binding affinities (*K*_A_) were determined by acquiring CEST images of phantom tubes containing 20 mM SRs and titrations of lactate concentrations between 0 and 600 mM at pH 6–7. The amplitude of the CEST effect was subsequently analyzed using a theoretical binding model outlined by Zhang et al.^8^ We assumed a single binding site for lactate in the model. The analysis enabled the determination of *K*_A_ and the maximum CEST effect at saturation (Figure 3A,B). Our findings revealed that all *K*_A_ values were ≤10^2^ M^–1^, underscoring a modest interaction between lactate and the SRs (Table 1). These results are particularly significant for translating SRs into noninvasive *in vivo* metabolic imaging applications in both preclinical and clinical settings. The determined *K*_A_ values indicate that lactate remains abundant in the bloodstream without being depleted by the SRs, confirming the feasibility of using these complexes for imaging purposes.

The detection of lactate with the SRs also proved to be specific in the presence of potentially confounding –OH and –NH resonances present in cell growth media (Dulbecco’s modified Eagle’s medium, DMEM). There were no notable differences in the CEST effect at 109 ppm when lactate-Yb-PCTA was combined with an equivalent amount of citrate, bicarbonate, or phosphate (Figure 3C). In the case of Eu-PCTA, neither the addition of these metabolites nor the presence of DMEM significantly affected the CEST intensity at 10–14 ppm. The only exception was citrate, which notably generated a significantly higher CEST effect (Figure 3D), but no CEST signal was observed for citrate-Eu-PCTA alone. The exchange rates for lactate-Eu-PCTA and citrate-lactate-Eu-PCTA were determined to be 3350 and 5610 s^–1^, respectively (pH 7, 21 °C, Figure 3E). This suggests that the difference in the CEST effect is not due to the citrate –OH CEST effect but rather arises from a proton catalyzed buffering effect.^32^ Recently, it has been reported that lanthanide complexes such as Eu-DO3A can also be used to detect inorganic phosphate.^33^ With the PCTA-based complexes, we did not observe any impact in the presence of 1 equiv of phosphate. While it has been broadly demonstrated that phosphates interact with lanthanide-based CAs,^34^ inorganic phosphate levels vary from 1.1 to 1.2 mM in serum^35,36^ and may increase to 1.8–2.5 mM in the microenvironment of some tumors.^37,38^ Lactate, on the other hand, accumulates in the TME at usually higher concentrations (2–12.9 mM).^39,40^ Therefore, we do not foresee a significant impact of phosphate on the detection of extracellular lactate in tumor tissue.

We also conducted lactate titrations in serum using both SRs. Results show a linear correlation between the CEST effect and lactate concentration (Figure S6C), indicating that the SR offered a robust and specific tool for measuring lactate in biological solutions. According to the Human Metabolome Database (hmdb.ca), lactate is present in blood at concentrations 10^2^–10^3^ times higher than those of other metabolites such as pyruvate, acetate, malate, succinate, and citrate under physiological conditions. Therefore, we expect the impact of these molecules on the detection of extracellular lactate with CEST to be negligible. We hypothesize that the apparent selective interaction between lactate and Ln-PCTA may be facilitated by its hydroxyl and carboxylate groups, which are less prone to steric hindrance effects compared to other carboxylic acids like citrate. Moreover, the –OH and –CO_2^–^_ groups may effectively coordinate and stabilize the Ln-PCTA complexes.^41^ The geometry and electronic configuration of the metal ion within the complex, along with the spatial rearrangement of the ligands, likely favor the selective binding of lactate over other monodentate anions (vide structural ^1^H NMR study in Figures 2A and S2–S5), as previously reported.^42,43^ Additionally, under pathological conditions such as cancer, the abundant presence of lactate in the TME likely enhances its preferential binding to Ln-PCTA.

To further test the potential of detecting extracellular lactate, we added 20 mM of the SRs to samples of MC-38 colon adenocarcinoma cell growth media collected after 48 h of culture in a normoxic incubator. Furthermore, we cultured cells with or without pyruvate to test if the SRs could quantify small changes in lactate excretion. Pyruvate is the precursor of lactate in the glycolytic pathway and is usually provided in the growing medium of fast-growing cancer cells. We acquired CEST images of tubes containing the media, 20 mM lactate, or no lactate after titrating the pH to 6 in all samples (16 μT, 5 s saturation pulses as shown in Figure 3F). Lactate concentrations were quantified using calibration curves based on CEST effects at 14 and 109 ppm. The values calculated from CEST images ranged between 19.9 and 25.1 mM, which closely aligned with the results from enzymatic assays (22.8 and 24.6 mM; Table 2). This strong correlation demonstrates that SRs and CEST MRI can accurately detect and quantify metabolic signatures in the TME, offering a reliable tool for profiling various cancer cell lines.

**Table 2:** Quantification of Lactate (mM) Excreted by MC-38 Cells with CEST MRI and the SRs^a^

	Pyr+	Pyr–
LDH Kit	24.6 ± 3.6	22.8 ± 3.1
Yb-PCTA CEST	25.1 ± 2.3	22.2 ± 2.9
Eu-PCTA CEST	24.8 ± 1.0	19.9 ± 0.9

a Lactate concentrations (mM) were determined with an enzymatic LDH kit and CEST MRI with 20 mM Yb- and Eu-PCTA in samples of MC-38 cells culturing medium. Cells were cultured for 48 h in the absence (Pyr−) or presence of 1 mM pyruvate (Pyr+). The CEST effect at 14 ppm (Eu-PCTA CEST) or 109 ppm (Yb-PCTA CEST) was used to calculate the lactate concentration based on a lactate-CEST calibration line.

To explore the feasibility of *in vivo* CEST imaging of extracellular lactate with the SRs, we tested the cytotoxicity of the SR *in vitro* and compared it with that of a Gd-based CA (Magnevist, Bayer) and Eu-DO3A. No significant differences in cytotoxicity between these complexes were observed, even at the high concentrations required for CEST imaging (millimolar range) (Figure S9). Furthermore, we incubated Gd-PCTA with lactate and performed titrations using up to 10 equiv of ZnCl_2_ or CaCl_2_, measuring *T*_1_ values over a 24 h period. The *T*_1_ values for the solutions containing ZnCl_2_ or CaCl_2_ varied by no more than ±1% compared to the samples without zinc/calcium, indicating that the Ln-PCTA complexes remain stable and unaffected by transmetalation, even in the presence of highly concentrated cations (Figure S10). This kinetic stability suggests that these complexes are suitable for *in vivo* applications.

**Figure 4:**
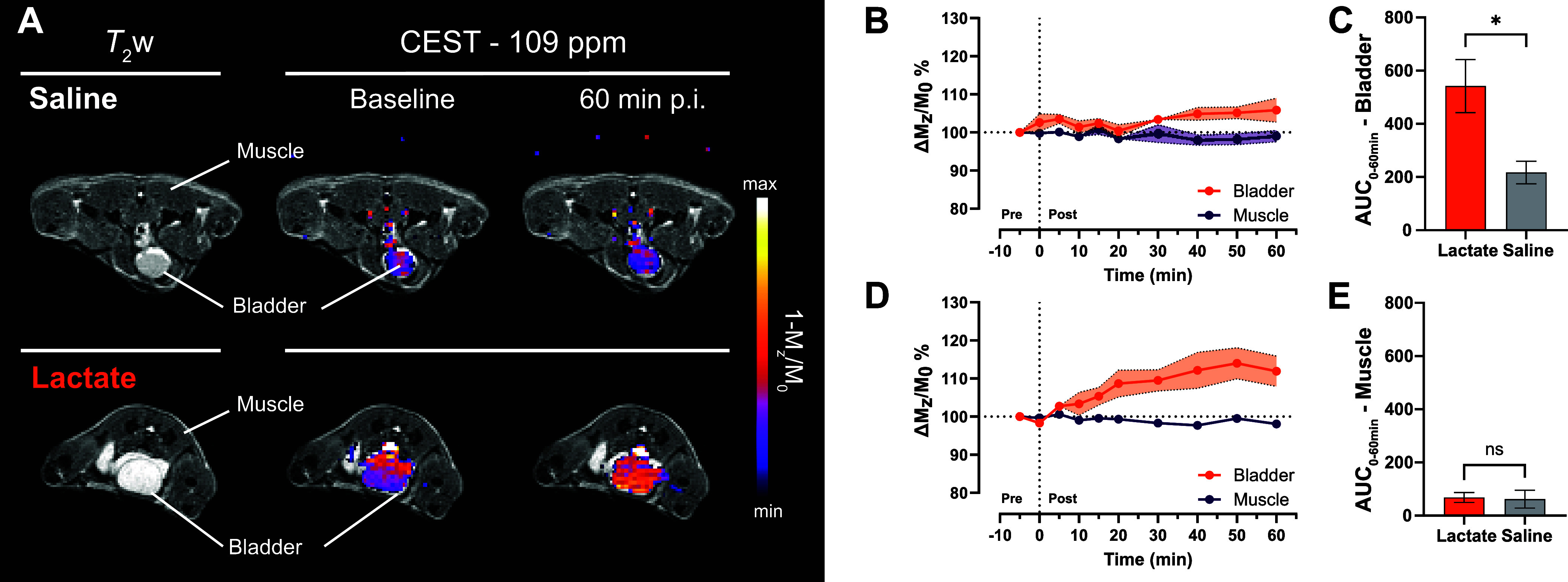
*In vivo* MR imaging of extracellular lactate with Yb-PCTA in healthy mice. (A) Representative axial anatomical (*T*_2_-weighted) and extracellular lactate (CEST 109 ppm) images of healthy C57BL/6 mice before and 60 min after the intravenous injection of 0.2 mmol/kg SR in saline solution or as a 1:1 mixture of lactate·Yb-PCTA. CEST images were acquired at a fixed offset (109 ppm) after 3 s presaturation pulses of 14 μT. (B,D) Quantification of the CEST effect in muscle tissue and bladder before (pre) and after (post) the injection of 0.2 mmol/kg Yb-PCTA 1:1 lactate·Yb-PCTA (D) or in saline solution (B). (C,E) Area under curve of the Δ*M*_*z*_/*M*_0_ % dynamics determined in bladder (C) and muscle (E). Data expressed as mean ± SEM (*n* = 3). *p < 0.05; ns, not significant (Student’s *t*-test).

Finally, we conducted *in vivo* studies using Yb-PCTA in healthy and tumor-bearing mice. First, we tested the feasibility of detection of shiftCEST produced by lactate-Yb-PCTA *in vivo*. We injected 0.2 mmol/kg of a 1:1 mixture of lactate-Yb-PCTA intravenously into 3 healthy C57BL/6 mice. Then, we acquired CEST images at a fixed saturation offset (109 ppm) to dynamically monitor the bladder and muscle tissue over 60 min, as reported previously.^44,45^ We observed a CEST effect, represented as *M*_*z*_ magnetization difference compared to the baseline (Δ*M*_*z*_/*M*_0_ %), in the bladder starting from 20 min after injection, reaching a maximum at 50 min (Figure 4A, D). This effect was not observed when we injected the same animals with Yb-PCTA dissolved in a 0.9% saline solution (Figure 4B). The area under curve of Δ*M*_*z*_/*M*_0_ % determined in the bladder over 60 min (AUC_0–60min_) was significantly higher in the animals injected with lactate (*p* = 0.040; Figure 4C). In the muscle, no significant difference was observed between the lactate and saline solution (*p* = 0.879; Figure 4E). The animal study demonstrated the safe *in vivo* detection of the ternary complex lactate-Yb-PCTA. Mice received two injections within 48 h and tolerated both well, indicating no acute toxicity associated with the intravenous administration of Yb-PCTA at this dose. The dynamics of the CEST effect in the bladder indicated that the SR was cleared quickly via renal elimination, with nearly complete clearance at 50 min postinjection (Figure 4C). This is in line with the typical biodistribution patterns of cyclen- and pyclen-based MR CAs. Previous reports showed that Ln-PCTA complexes are rapidly excreted, primarily by the kidneys (and to a lesser extent by the liver), with nearly complete clearance from the body within 2 h with no Gd^3+^ deposition observed in vital organs.^14,25^ Based on the moderate affinities of lactate to Yb-PCTA, we hypothesize that the CEST signal detected in the bladder is in equilibrium between the initial lactate provided with the injection and the lactate secreted from the tissues. Nevertheless, we could not determine whether the lactate-Yb-PCTA ternary complex was excreted intact or if the complex was reconstituted in the urine—an acknowledged limitation.

**Figure 5:**
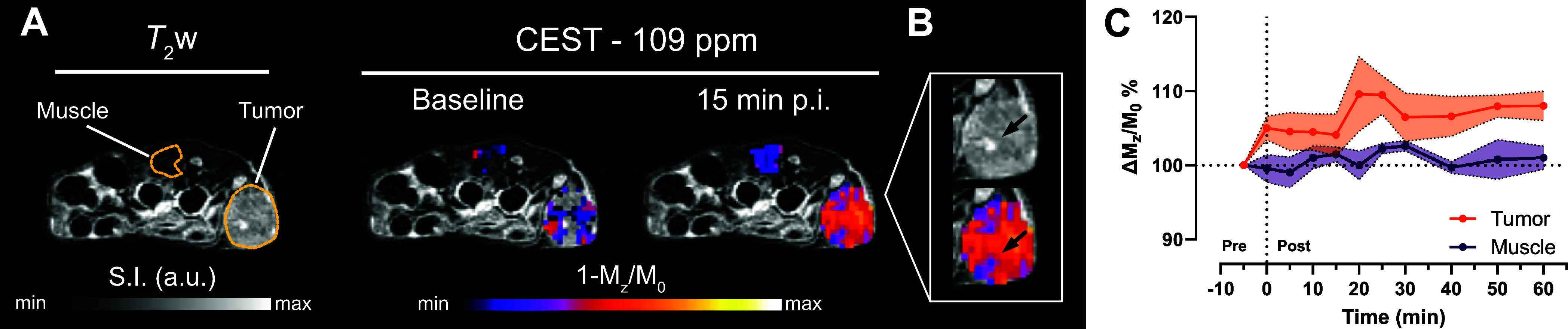
*In vivo* MR imaging of extracellular lactate with Yb-PCTA in MC-38 tumor-bearing mice. (A) Representative axial anatomical (*T*_2_-weighted) and extracellular lactate (CEST—109 ppm) images acquired before (baseline) and 15 min after a bolus injection of 1.0 mmol/kg of Yb-PCTA. (B) Zoomed view of an edematous spot within the tumor (black arrows) in the anatomical (top) and CEST images (bottom). (C) Quantification of the CEST effect in tumor and muscle tissue before (pre) and after (post) the injection (*n* = 4; mean ± SEM).

In another *in vivo* experiment, we applied the same dynamic CEST MRI protocol to tumor-bearing mice. We inoculated MC-38 colon adenocarcinoma cells subcutaneously in the lower flank of C57BL/6 mice. To account for the reduced perfusion of the tumors, we increased the dose of Yb-PCTA to 1 mmol/kg. We observed CEST contrast in the tumor region immediately after injection (Figure 5A,C), while no contrast was detected in the muscle tissue throughout the 60 min scan. The magnitude of the effect was heterogeneous within the tumors and smaller than that observed in the bladder, likely due to the uneven distribution of Yb-PCTA across the tumor and the heterogeneous accumulation of lactate in the TME. Areas of poor perfusion, such as edematous regions, showed reduced CEST signals (Figure 5B). These experiments demonstrated that Yb-PCTA CEST MRI can effectively track extracellular lactate distribution in the TME. Additionally, they confirmed that Yb-PCTA can be administered intravenously at high doses with no apparent acute toxicity. Our results demonstrate the feasibility of detecting lactate using CEST MRI with Ln-PCTA complexes; nevertheless, the *in vivo* validation has some limitations due to the small number of animals used in the preclinical study. To evaluate the full translational potential, further work should focus on thoroughly understanding the biodistribution and biosafety of the SRs and evaluating the effectiveness of this approach in additional disease models. Additionally, while the study demonstrates a robust CEST signal for lactate with Ln-PCTA complexes *in vitro*, the sensitivity of CEST MRI for detecting extracellular lactate *in vivo* may be influenced by the tissue vascularity, perfusion, and motion effects. Ratiometric approaches to quantify the CEST effect using chiral SRs, or the coinjection of a radiolabeled and nonradiolabeled Ln-PCTA complex, could be explored to overcome this limitation. Improving the CEST signal-to-noise ratio with optimized CEST sequences to reduce motion artifacts will also aid in achieving quantitative metabolic imaging.^46,47^

## Conclusions

Our study showed that Ln-PCTA complexes can form ternary complexes with lactate. High-resolution NMR analysis successfully demonstrated the interaction between these complexes and lactate. Remarkably, Yb-PCTA and Eu-PCTA showed only a TSD isomer structure in solution at several temperatures when coordinated with lactate.^22,23^

We assessed the CEST effect and its specificity for lactate under various conditions. Yb-PCTA and Eu-PCTA exhibited strong and unique CEST signals in the presence of lactate, supporting their potential as effective CEST SRs. The chemical structure, the typical pathophysiological concentrations, and cellular localization of other potentially competing metabolites make them less favored to interact with the SRs. The *K*_A_ values indicated a moderate binding between lactate and the SRs, suggesting that physiological lactate levels remain stable in the bloodstream during the use of these SRs for *in vivo* molecular imaging. Our experiments in cell culture media and *in vitro* cytotoxicity assessments demonstrated that these SRs are safe and suited for noninvasive *in vivo* imaging. Furthermore, we validated the safety and efficacy of detection *in vivo*. The preclinical *in vivo* experiments demonstrated that a 0.2 mmol/kg dose of Yb-PCTA—similar to the clinical dose of Gd-based CAs and much lower than that of typical iodine CT agents—could be detected intact in the mouse bladder, where most of the injected dose is excreted. Additionally, we detected extracellular lactate in poorly perfused subcutaneous tumors in mice using a higher dose. While a robust detection of extracellular lactate in tumors or organs outside the main excretion routes may require high SR doses, ongoing optimization of the CEST acquisitions and the *k*_ex_ and *K*_A_ of lactate-Ln-PCTA complexes may help compensate for the moderate CEST SNR generated. This optimization could potentially enhance the CEST effect, allowing for lower injection doses and weaker presaturation pulses for CEST detection. Further thorough analysis of the biosafety will then be needed for a translation of the results.

The findings of this study provide valuable insights for developing noninvasive metabolic imaging probes in preclinical and clinical settings. This study demonstrates the potential of combining CEST MRI with safe inorganic lanthanide-based PCTA complexes to effectively characterize different cancer cell lines and the metabolic tumor microenvironment.

## Methods

### Chemicals and Synthesis

1.0015 g of pyclen was added to a 100 mL flask. 5.4043 g of K_2_CO_3_ was added (8.2 equiv). About 34 mL of acetonitrile was added. The slurry was stirred while cooling. After reaching 5 °C, 2.03 mL of *t*-butyl bromoacetate was added straight and stirred overnight. The solution was allowed to warm to room temperature and then was stirred overnight. The solution was filtered to remove the salts and then split into two separate vessels. An equivolume of water was added to each. 1.8 mL of 1 M HCl was added to lower the pH from 11.3 to 3–4. An equivalent of 0.5 M EuCl_3_, YbCl_3_, or PrCl_3_ was added. The Eu and Yb solutions were heated to 45–55 °C for complexation. The Pr solutions were heated to 75 °C for complexation. Throughout the day to form the complex, 1 M NaOH was used to maintain pH = 4–6 (Eu^3+^), pH = 5.3–5.6 (Yb^3+^), and pH = 5.8–6.5 (Pr^3+^). Acetonitrile was replenished as needed to maintain a consistent volume. The complexation was monitored by HPLC. After complexation, the metal acts as a catalyst to remove the *t*-butyl esters. The reactions were stopped after >80% completion. The solutions were used as a stock solution for preparative HPLC purification. A Phenomenex Luna C18(2) column connected to a Waters DeltaPrep system was used. A simple gradient using 0.025% TFA in CH_3_CN/H_2_O modifiers was used. Collected fractions were freeze-dried to obtain the purified complexes. Final purities were ≥98% by HPLC. Identities were verified by mass spectrometry. The solids were analyzed by ICP–MS to quantify the metal content: 21.8% Eu in Eu-PCTA, 24.9% Yb in Yb-PCTA, and 17.4% Pr in Pr-PCTA. Based on the percent metal values, it is likely the compounds were isolated as a 1TFA salt with a small percentage of residual water. Isolation of a 1TFA salt was observed in our laboratory with similar complexes. Percent yields are based on the formula weight of the 1TFA salt. Synthetic quantities, molar equivalents, and chemical yields are shown in Table S1 from the Supporting Information. Chemical structures and IUPAC names were obtained using Chemaxon MarvinSketch 24.1.2.^48^

### Permeability Artificial Membrane Penetration Assay

The PAMPA was performed as previously described.^21^ An artificial membrane was produced in a donor plate (MAIPNTR10 PDVD, Merck) by adding lecithin (l-α-phosphatidylcholin, lecithin, Merck) in dodecane (Merck) solution (1% w/v). To assess the membrane permeability of the compounds, 200 μL of 1 mM Ln-PCTA complexes in PBS was pipetted into each donor well of the PAMPA plate. The wells of the acceptor plate (96-deepwell plate, Thermo Scientific) were each filled with 1100 μL PBS. The donor plate was placed into the acceptor and then the plates were left at room temperature for 21 h, after which the contents of the acceptor wells were analyzed by HPLC and quantified using a calibration curve. Propranolol was used as a positive control. The assay was performed in triplicate (Eu-PCTA and propranolol: duplicate).

### NMR Experiments

NMR measurement of the BMS of cell growing medium containing Yb-PCTA or Gd-DOTA was performed by measuring the chemical shift of *t*Bu as previously described.^49^ PyMT-derived ML1B1B1 breast cancer cells were cultured as described in “Cell Culture”. For experiments, cells were seeded at a density of 5 × 10^3^/well in 96-well plates. After 24 h, at 90% confluence, growing medium was removed, and cells were incubated with DMEM containing 2 mM Yb-PCTA or Gd-DOTA (Dotarem, Guerbet) for 2 h. Wells containing no cells were processed in the same way and were used as negative control. After 2 h, the growing medium was collected, centrifuged at 10 g for 5 min, and stored at 4 °C until further analysis. For the BMS NMR measurements, samples of the growing medium were mixed with 10% *t*Bu and transferred to 5 mm NMR tubes. The concentration of Yb-PCTA and Gd-DOTA was derived from the chemical shift of *t*Bu –OH peak as previously described.^49^ The concentration of the lanthanide complexes was derived by the chemical shift of *t*Bu and compared with that of wells without cells.

NMR solutions to analyze the interaction between lactate and SRs were prepared using deuterated solvent D_2_O. The ^1^H NMR spectra were acquired on a Bruker AVANCE III 400 spectrometer (Bruker, Massachusetts, USA) operating at a frequency of 400.13 MHz (^1^H), at various temperatures, using a 5 mm z-gradient inverse probe (Figures S4–S6). 2D COSY and EXSY spectra were acquired on a Bruker AVANCE NEO 600 spectrometer, using BBFO 5 mm iProbe (Figure S7). Subsequently, the resulting data were processed and analyzed using Topspin v4.0 (Bruker) and MestReNova 9.1 (Mestrelab).

### CEST MRI Experiments

Phantoms were acquired by using a 7 T (300 MHz) preclinical MRI scanner (Bruker BioSpec 70/30, Bruker BioSpin, Ettlingen, Germany) using an 86 mm diameter ^1^H transceiver volume coil (Bruker). 2D CEST coronal images were acquired using a previously reported FISP sequence with the following parameters:^50^ echo time (TE) 1.80 ms, repetition time (TR) 3.60 ms, flip angle 30°, field of view (FOV) 100 × 80 mm, slice thickness 1 mm, matrix size 128 × 80, resolution 0.78 × 0.75 × 1 mm. CEST presaturation consisted of 5 s, continuous rectangular pulses with a B_1_ ranging between 2 and 21 μT depending on the experiment. Different sets of saturation offsets were used depending on the Ln-PCTA. Phantoms consisting of 0.3 mL Eppendorf tubes were placed in a customized 3D-printed phantom holder, filled with 2% agarose in order to reduce B_0_, B_1_, and temperature fluctuations.

### CEST Spectra

Z-spectra were acquired in phantom tubes containing 50 mM of Yb- and Eu-PCTA (Pr-PCTA: 40 mM) mixed with 50 mM lactate (for Pr-PCTA: 40 mM) in 50 mM HEPES buffer at pH 6 and 7, 298 K. Tubes without lactate with Ln-PCTA at the same concentrations, pH, and buffer concentration were used as controls. 5 s presaturation pulses were used with different B_1_ values depending on the experiment. Raw spectra were fitted to Lorentzian line shapes based on a two-pool model (water, lactate·Ln-PCTA) using an in-house-written MATLAB script. CEST effect with respect to saturation offset (Δω) was quantified as MT ratio asymmetry percentage (MTR_asym_ %), as per MTR_asym_ % = [(*M*_*z*_^–Δω^ – *M*_*z*_^+Δω^)/*M*_0_] × 100, unless differently specified.

### Calibration Curves for Lactate Determination with CEST MRI

Phantoms containing 20 mM SRs mixed with 0–40 mM lactate in 50 mM HEPES buffer or human serum (Sigma-Aldrich) at pH 6 and 7, 298 K were used to generate calibration curves between the amplitude of CEST effect at 14 ppm (Eu-PCTA) or 109 ppm (Yb-PCTA) and lactate concentration (Figure S6). CEST presaturation consisted of 5 s, 16 μT continuous pulses. Z-spectra were fitted to Lorentzian line shapes (two pools: water and lactate·Ln-PCTA). CEST images were acquired in triplicate.

### Exchange Rates Determination

Exchange rates were determined using the Omega plot method.^31^ 20 mM, 1:1 solution of lactate, and SRs at pH 6 or 7 were scanned as described before using CEST presaturation pulses with B_1_ ranging between 2 and 20 μT.

### Binding Affinity Determination

Binding affinity for the SRs–lactate complexes was determined using the CEST_%_ effect at 14 ppm (Eu-PCTA) or 109 ppm (Yb-PCTA) at pH 6 and 7, 298 K. CEST_%_ was determined as per CEST % = [1 – (*M*_*z*_^Δω^/*M*_0_)] × 100, using 20 mM SRs and lactate concentrations ranging between 0 and 600 mM. After background correction, CEST_%_ for each lactate concentration was fitted according to a previously reported equation to determine *K*_A_.^8^

### Competition Experiment

Selectivity of the CEST effect for lactate was tested in phantoms containing 20 mM SRs and different combinations of 40 mM lactate, citrate, NaHCO_3_, NaH_2_PO_4_, and DMEM at pH 7, 298 K. CEST images were acquired as described above with 5 s presaturation pulses with a B_1_ of 16 μT. Exchange rates were determined with the Omega plot as described in “Exchange rates determination”.

### Relaxometry Experiments

To determine *r*_1_ relaxivity, 0.1–1 mM Eu-PCTA phantoms and 0–10 mM Yb-PCTA, dissolved in human serum, were prepared in 0.3 mL tubes. *T*_1_ maps were acquired by using a standard 2D RARE VTR sequence with 15 TRs. Experiments were performed at 310 K in human serum. *T*_1_ maps were generated by using the MRI Analysis Calculator for ImageJ (Fiji).

### Stability in the Presence of ZnCl_2_ and CaCl_2_

The effect of physiological cations on the detection of lactate was tested using Gd-PCTA as a surrogate for Yb- and Eu-PCTA. Phantoms containing 0.5 mM Gd-PCTA, 0.5 mM Gd-PCTA with 10 mM lactate, 0.5 mM Gd-PCTA, 10 mM lactate, and different amounts of ZnCl_2_ or CaCl_2_ (from 0.5 to 5 mM, corresponding to 1–10 equiv) were scanned at 7 T, 298 K to acquire *T*_1_ maps over 24 h. pH was titrated to 7. *T*_1_ maps were acquired and analyzed as described in “Relaxometry Experiments”. *T*_1_ values were plotted against the ZnCl_2_/CaCl_2_ concentration (Figure S10A,C). To monitor the trend of *T*_1_ values over time, *T*_1_ values were normalized to the first acquired *T*_1_ map, as per *T*_1_ change % = (*T*_1(*t*)_/*T*_1(*t*=0)_) × 100, and then divided by the *T*_1_ change % of the tube containing no ZnCl_2_/CaCl_2_ (Figure S10B,D).

### Cell Culture

PyMT-derived ML1B1B1 cells (donated by Dr. Sabrina Hoffmann) were cultured with DMEM supplemented with 10% FCS, 1% penicillin/streptomycin, l-glutamine, 1 mM sodium pyruvate, 1% MEM amino acids, and 10 mM. MC-38 cells (Kerafast) were cultured with DMEM supplemented with 10% FCS, 1% penicillin/streptomycin, l-glutamine, 1 mM sodium pyruvate, and 15 mM HEPES buffer. Cells were regularly tested for mycoplasma contaminations. For experiments, cells were trypsinized and counted with trypan blue and then processed differently according to the experiment.

### Cell Culture Experiment to Detect Lactate Excreted by Cancer Cells

MC-38 cells were cultured as described in “Cell Culture”. For experiments, cells were seeded at a density of 5 × 10^5^/well in 6-well plates in DMEM supplemented with or without 1 mM sodium pyruvate. After 48 h, the growing medium was collected and centrifuged for 30 min at 4 °C in 10 kDa filter tubes. For CEST measurements of lactate excreted by the cancer cells, the filtered growing media were transferred to 0.3 mL tubes, Yb- or Eu-PCTA was added to a final concentration of 20 mM, and pH was adjusted to 7.0; then, CEST images were acquired as described before using presaturation pulses of 5 s with a B_1_ of 16 μT. Lactate-CEST (1 – *M*_*z*_/*M*_0_ %) calibration lines were used to quantify the lactate concentration. An enzymatic LDH kit (Lactate Assay Kit II, Sigma-Aldrich) was used to cross-validate the lactate concentration. Lactate concentrations determined via the enzymatic kit and the CEST images are reported in Table 2.

### Cytotoxicity

Cytotoxicity of Yb-, Eu-, and Pr-PCTA complexes was evaluated using an MTS assay (Promega, Madison, WI, USA) and compared with the previously reported SR (Eu-DO3A) and Gd-DTPA (Magnevist, Bayer). MC-38 cells were cultured as described in “Cell Culture”. For experiments, cells were seeded at a density of 2 × 10^3^ cells/well in 96-well plates in triplicate. After 24 h, cells were incubated for 2 h with 5 mM Yb-, Eu-, Pr-PCTA, 5 mM Eu-DO3A, or 10 μM Magnevist dissolved in phenol red-free DMEM, and then the MTS reagent mix was added. After further incubation at 37 °C for 2 or 4 h, the absorbance at 490 nm was measured. Cytotoxicity was determined as viability versus control by comparing the absorbance of treated cells to control cells, expressed as % (Figure S9).

### Animal Experiments

Animal experiments with C57Bl/6 mice were conducted following German federal regulations on the use and care of experimental animals and approved by the local authorities (Regierungspräsidium Tübingen). A total of 7 (3 healthy and 4 tumor-bearing) female mice were used. Four mice were injected subcutaneously in the lower flank with 5 × 10^5^ MC-38 cells, and tumors were allowed to grow for 2 weeks. For the imaging experiments, mice were kept under anesthesia using isoflurane in pure oxygen (5% induction, 1.5% maintenance), and a catheter was placed in a tail vein.

### *In Vivo* Dynamic CEST MRI

Axial 2D CEST images were acquired with the same FISP sequence used for phantoms, with a spatial resolution of 0.6 × 0.6 × 1 mm. For the experiment with healthy mice, 20 preinjection CEST images were acquired at 109 ppm after a presaturation of 3 s, 14 μT continuous pulses to determine the baseline, and then further CEST images were acquired every 5 min for the first 30 min and at 40, 50, and 60 min after a bolus i.v. injection of 0.2 mmol/kg of Yb-PCTA dissolved in 0.9% saline solution. After 48 h, the experiment was repeated by injecting a 1:1 mixture of 0.2 mmol/kg lactate*Yb-PCTA. Twenty CEST images were acquired at each time point and averaged. The same imaging protocol was applied to MC-38 tumor-bearing mice. Tumor-bearing mice were injected with 1 mmol/kg of Yb-PCTA dissolved in 0.9% saline solution.

The dynamics of the CEST effect were determined for bladder and muscle tissue (healthy mice) and in tumor and muscle (tumor bearing mice), as Δ*M*_*z*_/*M*_0_ % = [1 + (*M*_*z*_^Δω^_(*t*=0)_/*M*_0_^Δω^_(*t*=0)_) – (*M*_*z*_^Δω^_(*t*)_/*M*_0_^Δω^_(*t*)_)] × 100. Area under curve of the CEST effect dynamics over 60 min (AUC_0–60 min_) was calculated with GraphPad PRISM 10.1.1 (GraphPad Software, Boston, MA USA). AUC_0–60min_ of bladder and muscle after injection of saline or lactate was compared with a two-tailed Student’s *t*-test, using GraphPad. A statistically significant difference was assumed for *p* < 0.05.

Anatomical *T*_1_-and *T*_2_-weighted images were acquired for coregistration of the CEST images (Supporting Information).

## Supporting Information Available

Additional detailed descriptions of all the experimental procedures and materials, complex synthesis, MRI sequences, PAMPA, BMS NMR, relaxometry and cytotoxicity results, and additional relevant ^1^H NMR and CEST spectra (PDF)

## Abbreviations

AUC: area under curve
BMS: bulk magnetic susceptibility
CEST: chemical exchange saturation transfer
DMEM: Dulbecco’s Modified Eagle Medium
DOTA: 1,4,7,10-tetraazacyclododecane-1,4,7,10-tetraacetic acid
DO3A: 1,4,7,10-tetraazacyclododecane-1,4,7-triacetic acid
MRI: magnetic resonance imaging
MTR_asym_ %: Magnetization transfer ratio asymmetry percentage
NMR: Nuclear magnetic resonance
PAMPA: permeability artificial membrane penetration assay
SAP: square antiprism
SR: shift reagent
PCTA: 3,6,9,15-tetraazabicyclo[9.3.1]pentadeca-1(15),11,13-triene-3,6,9-triacetic acid
TSAP: twisted square antiprism
TSD: twisted snub disphenoid.
