# Supplementary material for: Metabolic PCTA-Based Shift Reagents for the Detection of Extracellular Lactate Using CEST MRI
Source: JACS Au. 2025 Feb 10;5(2):779–90. doi: 10.1021/jacsau.4c01020 (PMC11862947; doi:10.1021/jacsau.4c01020)
Supplement: Supplementary file 1 — au4c01020_si_001.pdf [file au4c01020_si_001.pdf]

Metabolic PCTA-Based Shift Reagents for the Detection of Extracellular Lactate Using CEST MRI.

Remy Chiaffarelli, Pedro F. Cruz, Jonathan Cotton, Tjark Kelm, Mohammad Ghaderian, Slade Lee, Max Zimmermann, Carlos F. G. C. Geraldes, Paul Jurek, André F. Martins

Supplementary information

Contents

Chemicals and synthesis 1

Permeability Artificial Membrane Penetration Assay (PAMPA) 2

CEST MRI Experiments 3

CEST Spectra 3

Calibration curves for lactate determination with CEST MRI 3

Exchange rates determination 3

Binding affinity determination 4

Competition experiment 4

Relaxometry experiments 4

Stability in the presence of $ZnCl_2$ and $CaCl_2$ 4

Cell culture 4

Cell culture experiment to detect lactate excreted by cancer cells 5

Cytotoxicity 5

Animal experiments 5

***In vivo* dynamic CEST MRI** 5

References 6

Supplementary Figures 7

Chemicals and synthesis

Pyclen was dissolved in acetonitrile. Potassium carbonate was added as a base. The solution was cooled in an ice bath before adding t-butylbromoacetate. The solution was allowed to warm to room temperature and then stirred overnight. The solution was filtered to remove the salts and then split into two separate vessels. An equivolume of water was added to each. 1 M HCl was added to lower the pH to 3-4. An equivalent of 0.5 M Europium chloride, Ytterbium chloride, or Praseodymium chloride was added. The Eu & Yb solutions were heated to 45-55°C for complexation. The Pr solutions were heated to 75°C for complexation. Throughout the day to form the complex, 1 M NaOH was used to maintain a pH = 4-6 (Eu^{3+}), pH = 5.3-5.6 (Yb^{3+}), and pH = 5.8-6.5 (Pr). Acetonitrile was replenished as needed to maintain a consistent volume. The complexation was monitored by HPLC. After complexation, the metal acts as a catalyst to remove the t-butyl esters. The reactions were stopped after > 80% completion. The solutions were used as a stock solution for preparative HPLC purification.

A Phenomenex Luna C18(2) column connected to a Waters DeltaPrep system was used. A simple gradient using 0.025% TFA in CH_3CN/H_2O modifiers was used. Collected fractions were freeze-dried to obtain the purified complexes. Final purities were $\geq$ 98% by HPLC. Identities were verified by mass spectrometry. The solids were analysed by ICP-MS to quantify the metal content: 21.8% Eu in Eu-PCTA, 24.9% Yb in Yb-PCTA, and 17.4% Pr in Pr-PCTA. Based on the percent metal values, it is likely the compounds were isolated as a 1TFA salt with a small percentage of residual water. Isolation of a 1TFA salt has been observed in our lab with similar complexes. Percent yields are based on the formula weight of the 1TFA salt. Chemical structures and IUPAC names were obtained using Chemaxon MarvinSketch 24.1.2.[1]

Table S1.

Complex	Metal Content	Neutral Molecule Fw: % Metal	TFA salt Fw: % Metal	Yield
Eu-PCTA	21.8%	529.3 g/mol: 28.7%	643.4 g/mol: 23.6%	56%
Yb-PCTA	24.9%	550.4 g/mol: 31.4%	664.4 g/mol: 26.0%	48%
Pr-PCTA	17.4%	518.3 g/mol: 27.2%	632.4 g/mol: 22.2%	22%
Gd-PCTA	n/d	534.6 g/mol: 29.4%	648.7 g/mol: 24.2%	63%

Permeability Artificial Membrane Penetration Assay (PAMPA)

The Permeability Artificial Membrane Penetration Assay was performed as previously described.[2] An artificial membrane was produced in a donor plate (MAIPNTR10 PDVD, Merck) by adding (5 µL) lecithin (L-α-Phosphatidylcholin, lecithin, Merck) in dodecane (Merck) solution (1 % w/v). The addition of the lecithin solution was accompanied by a visible wetting of the membrane. To assess the membrane permeability of the compounds, 200 µL of 1 mM Ln-PCTA complexes in PBS was pipetted into each donor well of the PAMPA plate. The wells of the acceptor plate (96 deepwell plate, Thermo Scientific) were each filled with 1100 µL PBS. The donor plate was placed into the acceptor plate and it was observed that full contact between the two was established. The plates were left at room temperature for 21 hours, after which the contents of the acceptor wells were analysed by HPLC and quantified using a calibration curve. The total system volume was 1300 µL, resulting in a theoretical equilibrium concentration of 153.8 µM. Propranolol, a positive control, has been extensively studied in such artificial membrane systems and shows high membrane permeability. As the propranolol was dissolved in DMSO, both donor and acceptor wells were formulated such that they both contained 1% DMSO. The assay was performed in triplicate (Eu-PCTA and propranolol: duplicate).

NMR Experiments

Nuclear magnetic resonance (NMR) measurement of the bulk magnetic susceptibility (BMS) of cell growing medium containing Yb-PCTA or Gd-DOTA was performed by measuring the chemical shift of tBu, as previously described.[3] PyMT-derived ML1B1B1 cells were were cultured as described in “Cell culture”. For experiments, cells were seeded at a density of 5×10^3/well in 96 well plates. After 24h, at 90% confluence, growing medium was removed, and cells were incubated with DMEM containing 2 mM Yb-PCTA or Gd-DOTA (Dotarem, Guerbet) for 2h. Wells containing no cells were processed in the same way, and were used as negative control. After 2h, the growing medium was collected, centrifuged at 10 g for 5 minutes, and stored at 4°C until further analysis. For the BMS NMR measurements, samples of the growing medium were mixed with 10% *t*Bu and transferred to 5 mm NMR tubes. The concentration of Yb-PCTA and Gd-DOTA was derived from the chemical shift of *t*Bu -OH peak as

previously described.[3] The concentration of the lanthanide complexes was derived by the chemical shift of tBu and compared to that of wells without cells.

NMR solutions to analyse the interaction between lactate and SRs were prepared using deuterated solvent D_2O. The ^1H NMR spectra were acquired on a Bruker Avance III 400 spectrometer (Bruker, Massachusetts, USA) operating at a frequency of 400.13 MHz (^1H), at various temperatures, using a 5-mm z-gradient inverse probe. For 1D data acquisition, the *zgpr* pulse sequence was implemented with 128 k complex points, a spectral width of 200000 Hz, a recycle delay of 0.05 s, and a 90° pulse width of 13.17 µs and 1024 transients (Figures S4-S6). 2D COSY and EXSY spectra were acquired on Bruker Avance NEO 600 spectrometer, using BBFO 5 mm iProbe (Figure S7). Subsequently, the resulting data was processed and analyzed using Topspin v4.0 (Bruker) and MestReNova 9.1 (Mestrelab).

CEST MRI Experiments

Phantoms were acquired using a 7 T (300 MHz) preclinical MRI scanner (Bruker BioSpec 70/30, Bruker BioSpin, Ettlingen, Germany) using an 86-mm diameter ^1H transceiver volume coil (Bruker). 2D CEST coronal images were acquired using a previously reported FISP sequence with the following parameters:[4] echo time (TE) 1.80 ms, repetition time (TR) 3.60 ms, flip angle 30°, field of view (FOV) 100x80 mm, slice thickness 1 mm, matrix size 128x80, resolution 0.78x0.75x1 mm. CEST pre-saturation consisted of 5 seconds, continuous rectangular pulses with a B_1 ranging between 2 and 21 µT depending on the experiment. Different sets of saturation offsets were used depending on the Ln-PCTA. Phantoms consisted of 0.3 mL Eppendorf tubes placed in a customised 3D-printed phantom holder, filled with 2% agarose in order to reduce B_0, B_1 and temperature fluctuations.

CEST Spectra

Z-spectra were acquired in phantom tubes containing 50 mM of Yb- and Eu-PCTA (Pr-PCTA: 40 mM) mixed with 50 mM lactate (for Pr-PCTA: 40 mM) in 50 mM HEPES buffer at pH 6 and 7, 298 K. Tubes without lactate with Ln-PCTA at the same concentrations, pH and buffer concentration were used as controls. 5 seconds pre-saturation pulses were used with different B_1 values depending on the experiment (B_1: 8, 12, 20 µT, Figure S8; B_1: 16 µT, Figure 2-3). Raw spectra were fitted to Lorentzian line shapes based on a two-pool model (water, lactate·Ln-PCTA) using an in-house written MATLAB script. CEST effect with respect to saturation offset (Δω) was quantified as Magnetization Transfer Ratio asymmetry percentage (MTR_{asym}%), as per $MTR_{asym}\% = [(M_z^{-\Delta\omega} - M_z^{+\Delta\omega})/M_0] \times 100$, unless differently specified.

Calibration curves for lactate determination with CEST MRI

Phantoms containing 20 mM SRs mixed with 0-40 mM lactate in 50 mM HEPES buffer or human serum (Sigma-Aldrich) at pH 6 and 7, 298 K, were used to generate calibration curves by obtaining linear regression lines between the amplitude of CEST effect at 14 ppm (Eu-PCTA) or 109 ppm (Yb-PCTA) and lactate concentration (Figure S6). CEST pre-saturation consisted of 5 seconds, 16 µT continuous pulses. Z-spectra were fitted to Lorentzian line shapes (two pools: water, and lactate·Ln-PCTA), and then the amplitude of the CEST effect (CEST%) was calculated with an in-house written MATLAB script. CEST images were acquired in triplicate.

Exchange rates determination

Exchange rates were determined using the Omega plot method.[5] 20 mM, 1:1 solution of lactate and SRs at pH 6 or 7 were scanned as described before using CEST pre-saturation pulses with B_1 ranging between 2 and 20 µT.

Binding affinity determination

Binding affinity for the SRs-Lactate complexes was determined using the $CEST_{\%}$ effect at 14 ppm (Eu-PCTA) or 109 ppm (Yb-PCTA) at pH 6 and 7, 298 K. $CEST_{\%}$ was determined as per CEST% = $[1-(M_z^{\Delta\omega}/M_0)] \times 100$. CEST images were acquired after a pre-saturation pulse of 5 seconds, 21 µT B_1, using 20 mM SRs and lactate concentrations ranging between 0 and 600 mM. After background correction, $CEST_{\%}$ for each lactate concentration was fitted according to the following equation to determine K_{A} and $CEST_{\%}$ at saturation ($CEST_{bound}$):[6]

$$CEST_{\%} = \frac{CEST_{bound} \cdot \left(NC_{EuDO3A} \cdot C_{EuDO3A} + C_{Lactate} + K_a^{-1} - \sqrt[2]{(NC_{EuDO3A} \cdot C_{EuDO3A} + C_{Lactate} + K_{\mathrm{A}}^{-1})^2 - 4 \cdot NC_{EuDO3A} \cdot C_{EuDO3A} + C_{Lactate}}\right)}{2 \cdot C_{EuDO3A}}$$

Competition experiment

Selectivity of the CEST effect for lactate was tested in phantoms containing 20 mM of SRs and different combinations of 40 mM lactate, citrate, $NaHCO_3$, NaH_2PO_4, Dulbecco's Modified Eagle's medium (DMEM) at pH 7, 298 K. CEST images were acquired as described above with 5 seconds pre-saturation pulses with a B_1 of 16 µT. Exchange rates were determined with the Omega plot as described in "Exchange rates determination".

Relaxometry experiments

To determine r_1 relaxivity, 0.1-1 mM Eu-PCTA phantoms and 0-10 mM Yb-PCTA, dissolved in human serum, were prepared in 0.3 mL tubes. T_1-weighted MR images of phantoms were acquired using a 2D T_1-FLASH sequence with the following parameters: TE 4 ms, TR 100 ms, flip angle 50°, field of view 50x50 mm, 10 slices, slice thickness 1 mm, matrix size 192x192, resolution 0.260x0.260x1 mm. T_1 maps were acquired using a standard 2D RARE VTR sequence with 15 TRs: 50, 100, 200, 300, 400, 500, 600, 700, 800, 900, 1000, 1500, 2500, and 3000 ms. Other parameters: TE 8 ms, field of view 50x50 mm, 2 slices, slice thickness 1 mm, matrix size 192x192, resolution 0.260x0.260x1 mm. Experiments were performed at 310 K in human serum. T_1 maps were generated using the MRI Analysis Calculator for ImageJ (Fiji).

Stability in the presence of $ZnCl_2$ and $CaCl_2$

The effect of physiological cations on the detection of lactate was tested using Gd-PCTA as a surrogate for Yb- and Eu-PCTA. Phantoms containing 0.5 mM Gd-PCTA, 0.5 mM Gd-PCTA with 10 mM lactate, and 0.5 mM Gd-PCTA, 10 mM lactate and different amounts of $ZnCl_2$ or $CaCl_2$ (from 0.5 to 5 mM, corresponding to 1-10 equivalents) were scanned at 7 T, 298 K to acquire T_1 maps over 24 hours. pH was titrated to 7. T_1 maps were acquired and analysed as described in "Relaxometry experiments". T_1 values were plotted against $ZnCl_2/CaCl_2$ concentration (Figure S10A ,S10C). To monitor the trend of T_1 values over time, T_1 values were normalised to the first acquired T_1 map, as per T_1 Change % = $(T_{1(t)} / T_{1(t=0)}) \times 100$, and then divided by the T_1 Change % of the tube containing no $ZnCl_2/CaCl_2$ (Figure S10B, S10D).

Cell culture

PyMT-derived ML1B1B1 cells (donated by Dr. Sabrina Hoffmann) were cultured in T175 flasks with Dulbecco's Modified Eagle's Medium (DMEM) supplemented with 10% FCS, 1% penicillin/streptomycin, L-glutamine, 1 mM sodium pyruvate, 1% MEM amino acids, and 10 mM HEPES in a humidified incubator (5% CO_2, 37°C). MC-38 cells (Kerafast) were cultured in T175 flasks with

DMEM supplemented with 10% FCS, 1% penicillin/streptomycin, L-glutamine, 1 mM sodium pyruvate, and 15 mM HEPES buffer in a humidified incubator (5% CO_2, 37°C). Cells were regularly tested for mycoplasma contaminations. For experiments, cells were trypsinized and counted with trypan blue, then processed differently according to the experiment.

Cell culture experiment to detect lactate excreted by cancer cells

MC-38 cells were cultured as described in "Cell culture". For experiments, cells were seeded at a density of 5×10^5/well in 6 well plates in a medium supplemented with or without 1 mM sodium pyruvate. After 48h, the growing medium was collected, centrifuged for 30 minutes at 4°C in 10 kDa filter tubes, and stored at -80°C until further use, while cells were harvested and counted with Tripan blue. For CEST measurements of lactate excreted by the cancer cells, the filtered growing media were transferred to 0.3 mL tubes, Yb- or Eu-PCTA was added to a final concentration of 20 mM, and pH was adjusted to 7.0. Tubes were transferred to a phantom holder filled with 2% agarose, then CEST images were acquired at room temperature as described before, using pre-saturation pulses of 5 seconds with a B_1 of 16 µT. Lactate-CEST (1-M_z/M_0 %) calibration lines were used to quantify lactate concentration. An enzymatic lactate dehydrogenase (LDH) kit (Lactate Assay Kit II, Sigma-Aldrich) was used to cross-validate lactate concentration. Lactate concentrations determined via the enzymatic kit and the CEST images are reported in Table 2.

Cytotoxicity

Cytotoxicity of Yb-, Eu-, and Pr-PCTA complexes was evaluated using an MTS assay (Promega, Madison, WI, USA) and compared to the previously reported shift reagent (Eu-DO3A) and Gd-DTPA (Magnevist, Bayer). MC-38 cells were cultured as described in "Cell culture". For experiments, cells were seeded at a density of 2000 cells/well in 96-well plates in triplicate. After 24 h, cells were incubated for 2 h with 5 mM Yb-, Eu-, Pr-PCTA, 5 mM Eu-DO3A or 10 µM Magnevist dissolved in phenol red-free DMEM, then the MTS reagent mix was added. After further incubation at 37°C for 2 or 4 h, absorbance at 490 nm was measured. Cytotoxicity was determined as viability versus control by comparing the absorbance of treated cells to control cells, expressed as %. Cytotoxicity of Ln-PCTA complexes was pooled and compared with that of Eu-DO3A and Magnevist (Figure S9).

Animal experiments

Animal experiments with C57Bl/6 mice were conducted following German federal regulations on the use and care of experimental animals and approved by the local authorities (Regierungspräsidium Tübingen). A total of 7 (3 healthy and 4 tumor-bearing) female mice were used. For MC-38 tumor inoculation, cells were cultured and counted as described in "Cell culture", then resuspended in serum free PBS. Four mice were injected subcutaneously in the lower flank with 5×10^5 cells and tumors were allowed to grow for 2 weeks. For the imaging experiments, mice were kept under anesthesia using isoflurane in pure oxygen (5% induction, 1.5% maintenance), and a catheter was placed in a tail vein. The mice were then placed in a heated mouse holder in the 7T MR scanner.

In vivo dynamic CEST MRI

Axial 2D CEST images were acquired with the same FISP sequence used for phantoms, with the following parameters: TE 1.76 ms, TR 3.52 ms, flip angle 30°, FOV 38.4x38.4 mm, slice thickness 1 mm, matrix size 64x64, spatial resolution 0.6x0.6x1 mm. For the experiment with healthy mice, 20 pre-injection CEST images were acquired at 109 ppm after a pre-saturation of 3 seconds, 14 µT continuous pulses to determine the baseline, then further CEST images were acquired every 5 minutes for the first

30 minutes and at 40, 50 and 60 minutes after a bolus i.v. injection of 0.2 mmol/Kg of Yb-PCTA dissolved in 0.9% saline solution. After 48 h, the experiment was repeated by injecting a 1:1 mixture of 0.2 mmol/Kg lactate*Yb-PCTA. 20 CEST images were acquired at each time point and averaged. The same imaging protocol was applied to MC-38 tumor bearing mice. Tumor bearing mice were injected with 1 mmol/Kg of Yb-PCTA dissolved in 0.9% saline solution.

The dynamics of the CEST effect were determined for bladder and muscle tissue (healthy mice) and in tumor and muscle (tumor bearing mice), as $\Delta M_z/M_0$ %= $[1+(M_z^{\Delta\omega}{}_{(t=0)}/M_0^{\Delta\omega}{}_{(t=0)}) - (M_z^{\Delta\omega}{}_{(t)}/M_0^{\Delta\omega}{}_{(t)})] \times 100$. Area under curve of the CEST effect dynamics over 60 minutes ($AUC_{0\text{-}60min}$) was calculated with GraphPad PRISM 10.1.1 (GraphPad Software, Boston, MA USA). $AUC_{0\text{-}60min}$ of bladder and muscle after injection of saline or lactate was compared with a two-tailed Student's t test, using GraphPad. A statistically significant difference was assumed for $p < 0.05$.

Anatomical 3D T_1 weighted- (FLASH sequence, TE 2.67 ms, TR 8.85 ms, flip angle 10°, matrix 272x136x96, FOV 76.8x34.8x22.8 mm, resolution 0.3x0.3x0.3 mm) and 3D T_2 weighted- (TurboRARE sequence, TE 90.51 ms, TR 1800 ms, flip angle 90°, matrix 256x116x76, FOV 75x35x21.12 mm, resolution 0.28x0.26x0.22 mm) images were also acquired before and 60 minutes after injection. Additionally, anatomical axial 2D T_2 weighted images (TurboRARE sequence, TE 33.58 ms, TR 5551 ms, flip angle 90°, matrix 128x128, 20 slices, FOV 38.4x38.4 mm, resolution 0.2x0.2x1 mm) were acquired for coregistration of the CEST images.

References

(1) *ChemAxon – Software for Chemistry and Biology R&D*. https://www.chemaxon.com/ (accessed 2017-04-26).

(2) Kansy, M.; Senner, F.; Gubernator, K. Physicochemical High Throughput Screening: Parallel Artificial Membrane Permeation Assay in the Description of Passive Absorption Processes. *J. Med. Chem.* **1998**, *41* (7), 1007–1010. https://doi.org/10.1021/jm970530e.

(3) Chu, K.-. C.; Xu, Y.; Balschi, J. A.; Springer JR, C. S. Bulk Magnetic Susceptibility Shifts in Nmr Studies of Compartmentalized Samples: Use of Paramagnetic Reagents. *Magn. Reson. Med.* **1990**, *13* (2), 239–262. https://doi.org/10.1002/mrm.1910130207.

(4) Sheth, V. R.; Li, Y.; Chen, L. Q.; Howison, C. M.; Flask, C. A.; Pagel, M. D. Measuring in Vivo Tumor pHe with CEST-FISP MRI. *Magn. Reson. Med.* **2012**, *67* (3), 760–768. https://doi.org/10.1002/mrm.23038.

(5) Dixon, W. T.; Ren, J.; Lubag, A. J. M.; Ratnakar, J.; Vinogradov, E.; Hancu, I.; Lenkinski, R. E.; Sherry, A. D. A Concentration-Independent Method to Measure Exchange Rates in PARACEST Agents. *Magn. Reson. Med.* **2010**, *63* (3), 625–632. https://doi.org/10.1002/mrm.22242.

(6) Zhang, L.; Martins, A. F.; Mai, Y.; Zhao, P.; Funk, A. M.; Clavijo Jordan, M. V.; Zhang, S.; Chen, W.; Wu, Y.; Sherry, A. D. Imaging Extracellular Lactate In Vitro and In Vivo Using CEST MRI and a Paramagnetic Shift Reagent. *Chem. Weinh. Bergstr. Ger.* **2017**, *23* (8), 1752–1756. https://doi.org/10.1002/chem.201604558.

Supplementary Figures

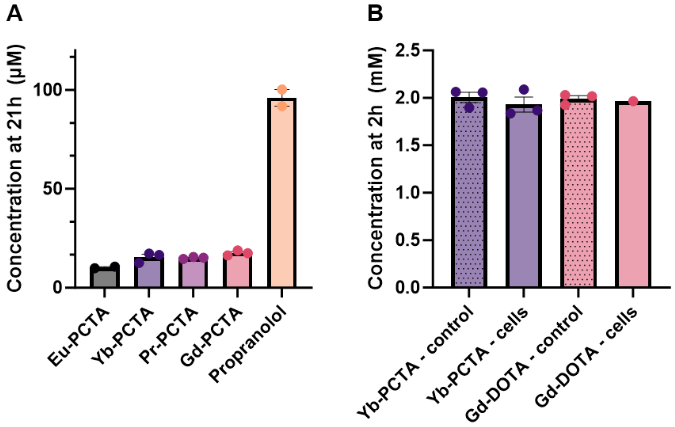

Figure S1. (**A**) Permeability Artificial Membrane Penetration Assay (PAMPA) of Ln-PCTA complexes. The Ln-PCTA complexes concentration in the acceptor plate was measured by HPLC and quantified using a calibration curve after an incubation of 21h at room temperature. (**B**) BMS NMR measurement of the concentration of Yb-PCTA and Gd-DOTA after 2h incubation in a 96 well plate containing PyMT ML1B1B1 or control wells (no cells). The ratio (%) of concentration between control and cells was 96.3% and 98.7% for Yb-PCTA and Gd-DOTA respectively.

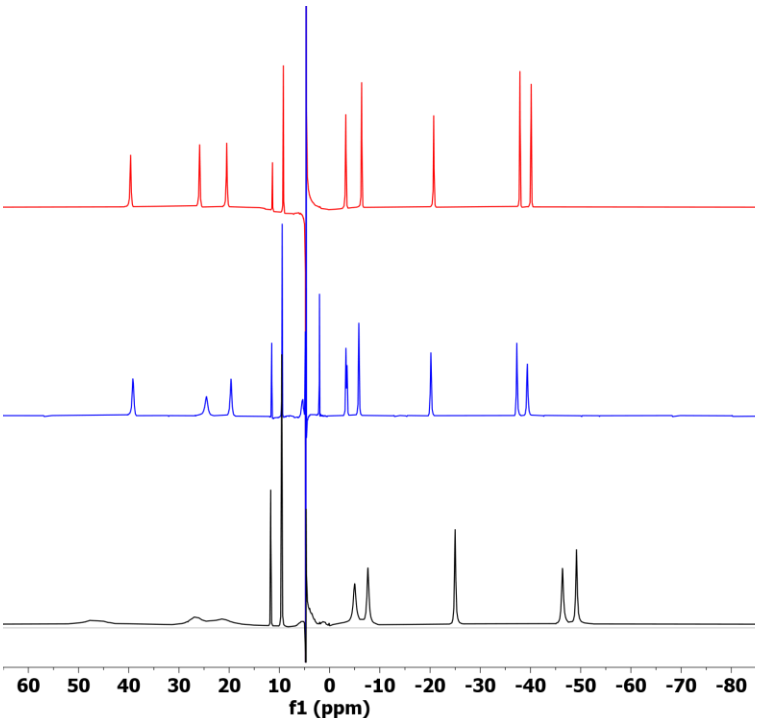

Figure S2. ^{1}H NMR spectra of Yb-PCTA at 298 K (black), 2:1 Lactate: Yb-PCTA mixture at 363 K (blue) and Yb-PCTA at 363 K (red)

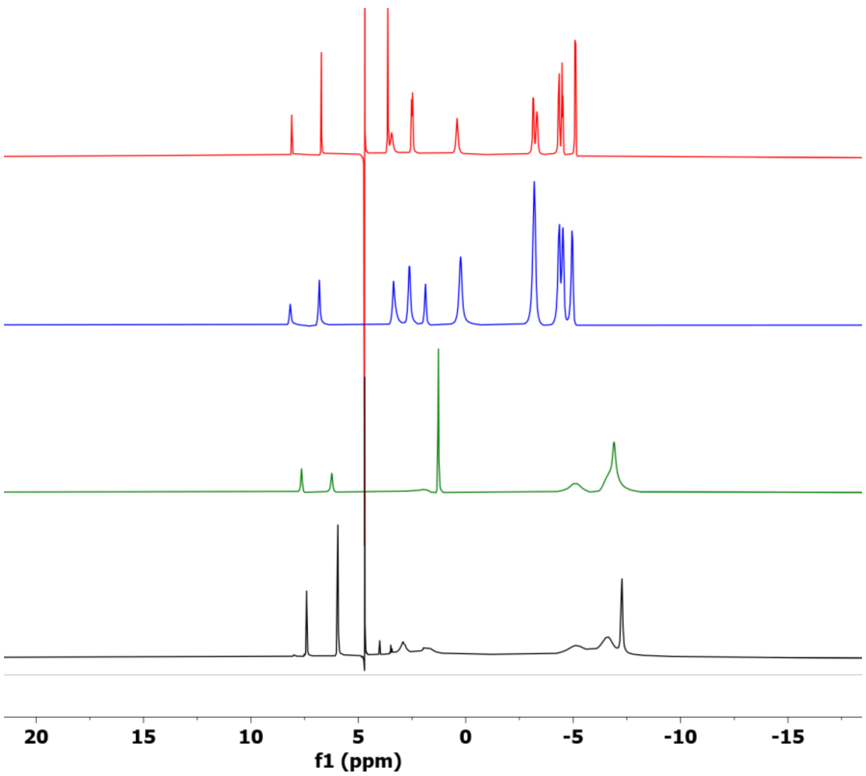

Figure S3. 1H NMR spectra of Eu-PCTA at 298 K (black), 2:1 Lactate: Eu-PCTA mixture at 298 K (green), 2:1 Lactate: Eu-PCTA mixture at 363 K (blue) and Eu-PCTA at 363 K (red).

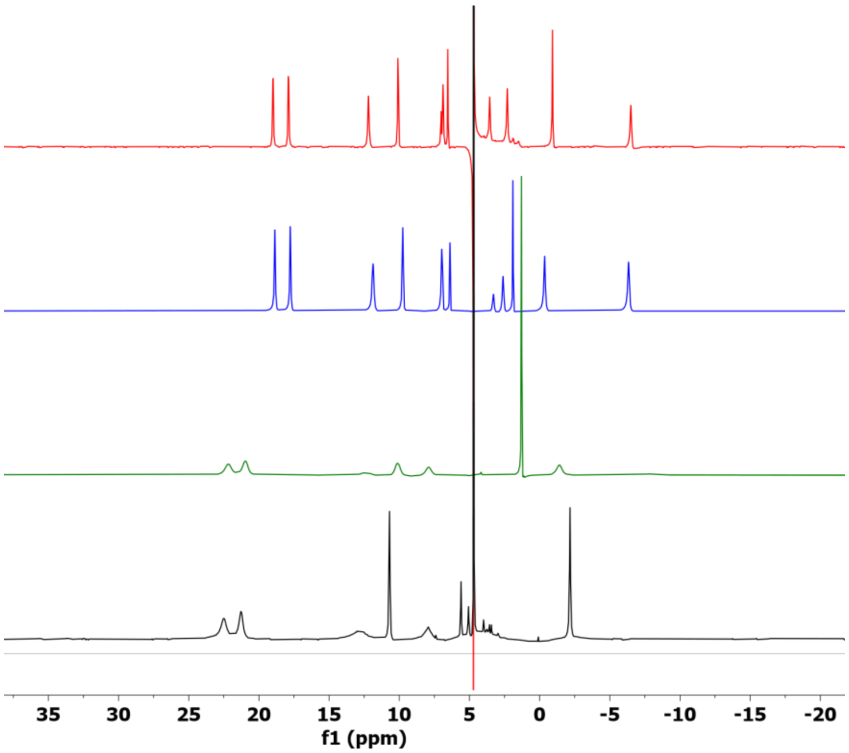

Figure S4. 1H NMR spectra of Pr-PCTA at 298 K (black), 2:1 Lactate: Pr-PCTA mixture at 298 K (green), 2:1 Lactate: Pr-PCTA mixture at 363 K (blue) and Pr-PCTA at 363 K (red).

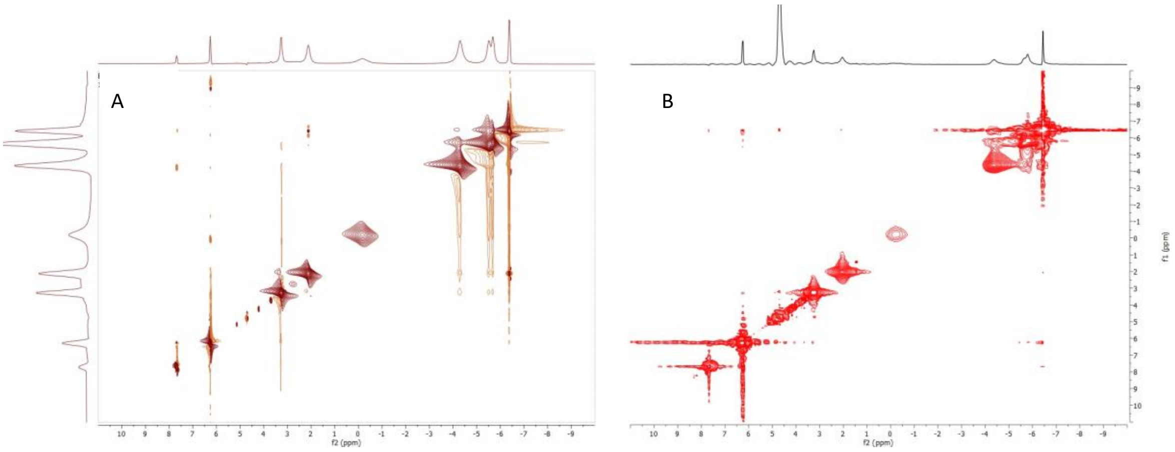

Figure S5. (A) 2D-COSY spectrum of an aqueous solution of Eu-PCTA at 298 K (pH 7.1). (B) 2D-EXSY spectrum of an aqueous solution of Eu-PCTA at 298 K (pH 7.1; mixing time, 10 ms).

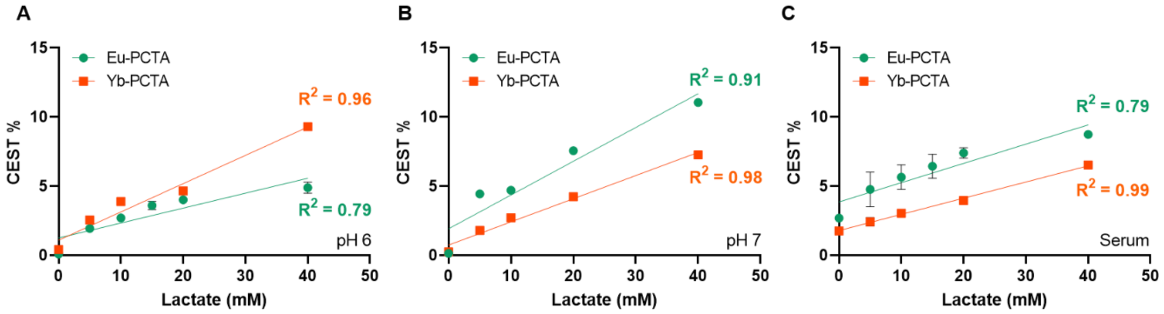

Figure S6. Plot of CEST effect at 14 ppm (Eu-PCTA) or 109 ppm (Yb-PCTA) vs lactate concentration, at pH 6 (A), pH 7 (B), or human serum at pH 7 (C). Goodness of the fit (R^2) of the linear regression analysis is reported for each set of lactate titration. Data expressed as mean CEST % ± SD of 3 scans.

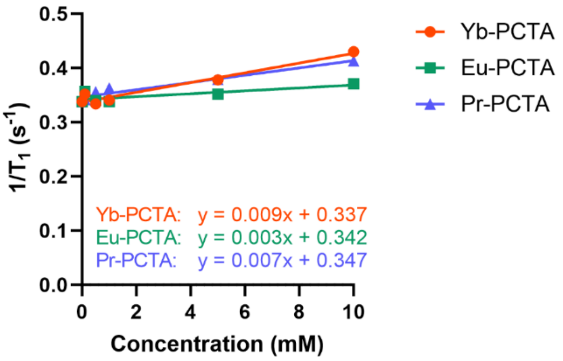

Figure S7. $1/T_1$ (s^{-1}) plot versus concentration (mM) of Yb-, Eu- and Pr-PCTA acquired in human serum at 310 K, at 7T. r_1 is the slope of the linear regression analysis.

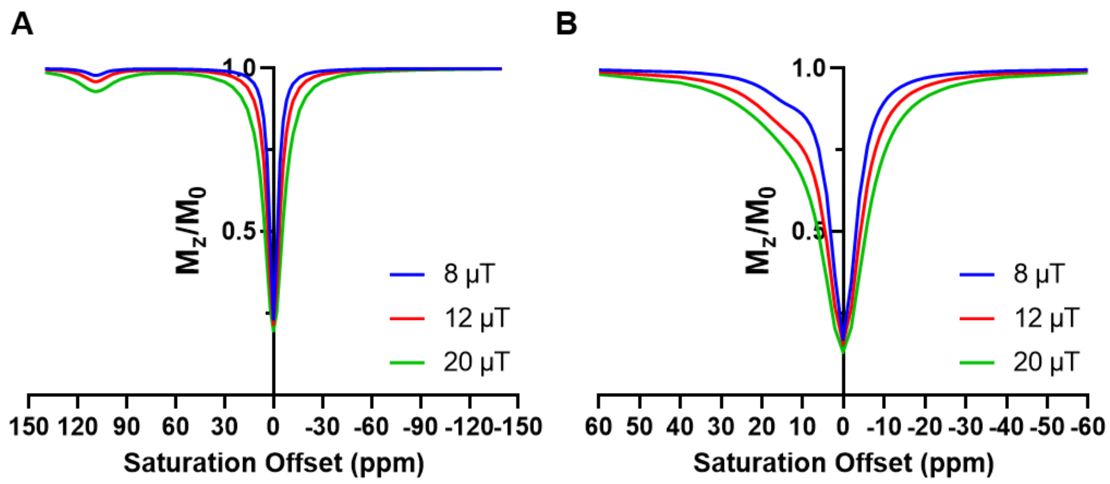

Figure S8. Z-spectra of 50 mM Yb-PCTA (A) or Eu-PCTA (B) mixed with 50 mM lactate acquired at 298 K, pH 6.9, with different B_1 values.

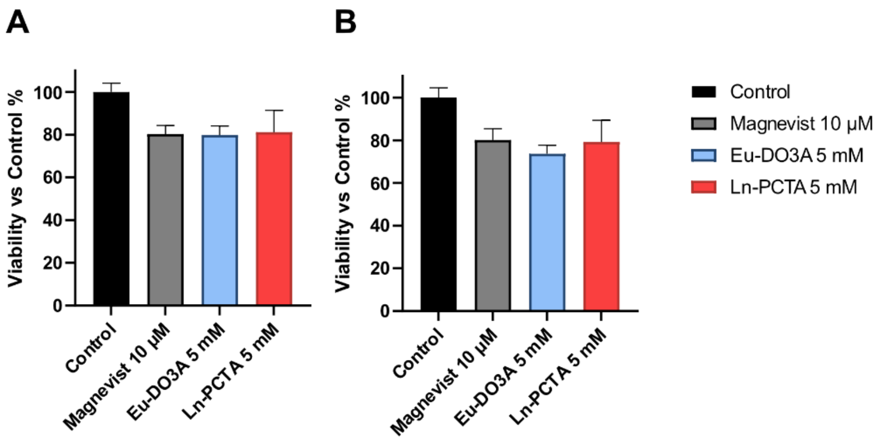

Figure S9. Cytotoxicity of Ln-PCTA complexes compared to Magnevist and Eu-DO3A. Cytotoxicity was determined with an MTS assay and calculated as percentage of viability compared to control cells (non-treated cells) after 2h (**A**) or 4h (**B**) of incubation. Data expressed as mean ± SEM (triplicates).

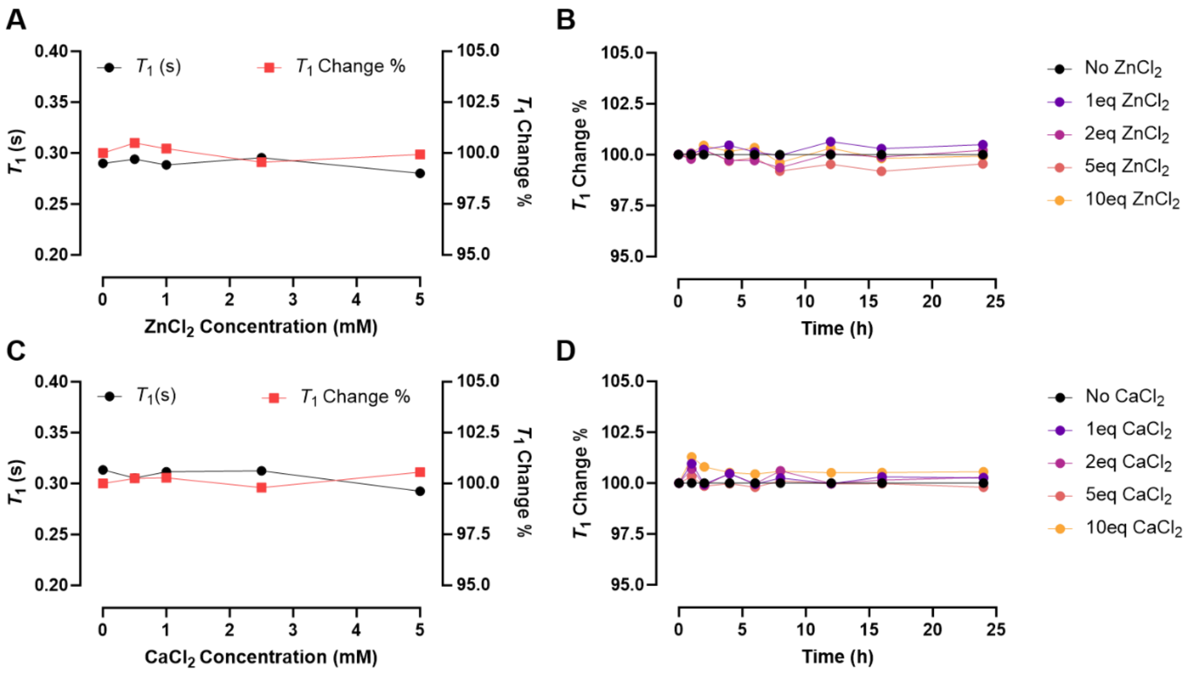

Figure S10. Stability of T_1 measurements in the presence of titrations of $ZnCl_2$ and $CaCl_2$in phantom tubes containing 0.5 mM Gd-PCTA and 10 mM lactate. (**A, C**) T_1 and T_1 Change % vs $ZnCl_2$ (**A**) or $CaCl_2$ (**C**) concentration, measured at 7 T, 298 K, pH 7, 24 hours after the phantom preparation. (**B, D**) T_1 Change % over time for phantom tubes containing 0.5 mM Gd-PCTA, 10 mM lactate and 0.5 mM Gd-PCTA, 10 mM lactate and different amounts of $ZnCl_2$ (**B**) or $CaCl_2$ (**D**) (ranging from 0.5 to 5 mM, corresponding to 1-10 equivalents). T_1 maps were acquired at 7 T, 298 K, pH 7, at different timepoints over 24 hours after the phantom preparation.
